# Effects of 3-Week Work-Matched High-Intensity Intermittent Cycling Training with Different Cadences on VO_2max_ in University Athletes

**DOI:** 10.3390/sports6040107

**Published:** 2018-09-29

**Authors:** Nobuyasu Tomabechi, Kazuki Takizawa, Keisuke Shibata, Masao Mizuno

**Affiliations:** 1 Sports Training Center, Nippon Sport Science University, Setagaya, Tokyo 158-8508, Japan; 2 Graduate School of Education, Hokkaido University, Sapporo, Hokkaido 060-0811, Japan; 3 Institute of Physical Development Research, Sapporo, Hokkaido 060-0061, Japan; takizawa@pd-r.org; 4 Department of Sustainable Agriculture, College of Agriculture, Food and Environment Sciences, Rakuno Gakuen University, Ebetsu, Hokkaido 069-0836, Japan; k-shibata@rakuno.ac.jp; 5 Faculty of Education, Hokkaido University, Sapporo, Hokkaido 060-0811, Japan; mizuno@edu.hokudai.ac.jp

**Keywords:** aerobic capacity, graded-exercise test, total workload

## Abstract

The aim of this study is to clarify the effects of 3-week work-matched high-intensity intermittent cycling training (HIICT) with different cadences on the VO_2max_ of university athletes. Eighteen university athletes performed HIICT with either 60 rpm (*n* = 9) or 120 rpm (*n* = 9). The HIICT consisted of eight sets of 20 s exercise with a 10 s passive rest between each set. The initial training intensity was set at 135% of VO_2max_ and was decreased by 5% every two sets. Athletes in both groups performed nine sessions of HIICT during a 3-week period. The total workload and achievement rate of the workload calculated before experiments in each group were used for analysis. VO_2max_ was measured pre- and post-training. After 3 weeks of training, no significant differences in the total workload and the achievement rate of the workload were found between the two groups. VO_2max_ similarly increased in both groups from pre- to post-training (*p* = 0.016), with no significant differences between the groups (*p* = 0.680). These results suggest that cadence during HIICT is not a training variable affecting the effect of VO_2max_.

## 1. Introduction

High-intensity intermittent (or interval) training (HIIT) is considered a time-efficient exercise strategy, due to its superior effect toward improving VO_2max_ in less time than low- or moderate-intensity continuous exercise [1,2]. In addition, HIIT can increase VO_2max_ in a short period (2–4 weeks) [3,4,5,6,7,8,9]. Since the total available time for training is often limited for athletes, it is important to determine the more effective HIIT methodology for increasing VO_2max_ in a short period.

HIIT with the use of a cycle ergometer is considered a safe method of training, and cadence may be a training variable that affects the chronic effect of VO_2max_. In previous studies, various training modes, such as cycling, running, aquatic treadmill running, jump rope, swimming, and kettlebell training, have been attempted [10]. Results showed that the stress on the anterior cruciate ligament was lower during cycling exercise [11,12]. In addition, cycling exercise was found to be associated with fewer eccentric contraction phases, which cause muscle damage, when compared to running [13]. Thus, HIIT by using a cycle ergometer can increase VO_2max_ more safely. The cadence used during work-matched cycling training may be a training variable that affects the chronic effect of VO_2max_ under relative intensity (e.g., %VO_2max_) and the length of time applied to the exercise-matched condition. The workload during cycling exercise is a product of load (kp) and cadence (rpm). Therefore, high-intensity intermittent cycling training (HIICT) can be performed either with high load/low cadence or with low load/high cadence under the workload, relative intensity (e.g., %VO_2max_), and exercise time-matched conditions [14]. Many previous studies reported that oxygen uptake (VO_2_) during work-matched cycling exercise increases more significantly in high-cadence cycling than in low-cadence cycling (35–110 rpm) due to the elevated internal workload of active muscles [15,16,17,18,19,20]. Thus, work-matched cycling exercise with a high cadence may have a higher actual intensity than cycling exercise with a low cadence, even though the relative intensity (e.g., %VO_2max_) is equal. Matsuo et al. reported that high-intensity interval training improves VO_2max_ more significantly than moderate-intensity training due to an increase in left ventricular mass and stroke volume [21]. Therefore, it can be speculated that HIICT with a high cadence can improve VO_2max_ more significantly than HIICT with a low cadence. In contrast, Paton et al. reported that high-intensity interval training with a low cadence significantly improved VO_2max_ compared to that of a high cadence in male cyclists [22]. However, workload was not matched in this study. Since the workload affects the chronic effect of VO_2max_ [23], the effect of the difference in cadence on VO_2max_ should be examined under work-matched conditions. 

The aim of this study, therefore, was to examine whether 3-week work-matched HIICT with a high cadence (120 rpm) can significantly improve VO_2max_ compared to HIICT with a low cadence (60 rpm) in university athletes.

## 2. Materials and Methods

### 2.1. Experimental Design

Participants were assigned to one of two groups according to their workload of HIICT, calculated based on pre-training VO_2max_. One group of participants performed HIICT with a low cadence of 60 rpm (*n* = 9, age: 20.1 ± 0.8 years, height: 174.6 ± 4.8 cm, body weight: 65.4 ± 3.9 kg) and the other group of participants performed HIICT with a high cadence of 120 rpm (*n* = 9, age: 20.0 ± 1.0 years, height: 173.2 ± 5.3 cm, body weight: 64.4 ± 6.3 kg). HIICT was performed by both groups in nine sessions during a 3-week period and at least twice per week in order not to bias the number of sessions per week. In both groups, training load was increased by 2.5% after every three sessions. All training sessions were supervised by investigators with expert knowledge of HIICT. VO_2max_ measurement during the graded-exercise test using a cycle ergometer was carried out pre- and post-training. All measurements for each participant were performed at approximately the same time of day (±2.5 h) to take into consideration the circadian rhythm.

### 2.2. Participants

A total of 21 Japanese male university athletes were initially recruited. However, three participants could not complete the training due to injuries unrelated to the experiment. Thus, data from 18 participants were used for further analysis. All participants practiced exercise at least twice per week and belonged to the university volleyball (*n* = 8), soccer (*n* = 3), soft tennis (*n* = 3), ultimate (*n* = 2), badminton (*n* = 1), and sailing (*n* = 1) teams. Participants did not habitually perform any physical training, except for practice for their respective sports; furthermore, no participants had performed resistance training for the lower body more than two times per week during the previous 6 months or performed any cycling training for a competitive race. All participants were informed about the potential risks of experiments and provided written consent prior to participation. This study was approved by the Ethics Committee of Faculty of Education, Hokkaido University (approval number: 17–24).

### 2.3. VO_2max_

The graded-exercise test using a cycle ergometer (Powermax-VII, Combi Wellness, Tokyo, Japan) was performed to determine VO_2max_ and relative intensity of the HIICT. The test was initiated at 60 W, followed by 30 W increases every 3 min until each participant was unable to maintain a cadence of 60 rpm. The cadence during the test was controlled by a metronome and was displayed on a screen. During the test, VO_2_ was measured every 10 s using mixing chamber methods with a respiratory gas analyzer (VO2000, S&ME Co. Ltd., Tokyo, Japan) and the peak value was defined as VO_2max_ [24,25,26]. 

### 2.4. High-Intensity Intermittent Cycling Training 

In all training sessions, the HIICT was performed by using a cycle ergometer (Powermax-VII, Combi Wellness, Tokyo, Japan) following a warm-up at 90 W for 10 min and a rest period of 3 min. The initial training intensity of the HIICT was set at 135% of VO_2max_ and was decreased by 5% every two sets. HIICT consisted of eight sets of 20 s pedaling, with a 10 s passive rest between each set. This protocol was conducted according to the results of our pilot study that was, in turn, based on previous studies [3,27,28]. Participants were instructed to maintain a cadence of either 60 rpm or 120 rpm, which was controlled by the value displayed on the screen and a metronome during each session. After the HIICT, participants performed a cool down at 90 W for 5 min in all training sessions. The total workload and achievement rate of the workload calculated before experiments involving each group were used for analysis. The cadence was decided to be insufficient if the workload during the 3-week period did not reach 90% of the workload calculated prior to the experiments; these data were excluded from the analysis. The average value of the absolute load of the HIICT during the training period is shown in Table 1. 

### 2.5. Statistical Analyses

All data are presented as means and standard deviations (SD). Total workload, achievement rate, baseline VO_2max_ levels, and percent change of VO_2max_ in both study groups were analyzed using the unpaired *t*-test. Moreover, the changes in VO_2max_ and body weight from pre- to post-training were analyzed by two-way (group × time) mixed-design analysis of variance (ANOVA; between-participant factor: group, within-participant factor: time). A post hoc analysis was performed using the Bonferroni test. The statistical significance level was set at *p* < 0.05. As indices of the effect size, Cohen’s d (for unpaired *t*-test and post hoc comparisons) and partial η^2^ (for ANOVA) were also calculated. SPSS Statistics (version 24.0 for Windows, SPSS Inc., Chicago, IL, USA) was used for data analysis. 

## 3. Results

All 18 participants who completed the nine training sessions exceeded the 90% achievement rate of the workload calculated prior to the experiments. No significant differences in the baseline VO_2max_ levels were found between the groups (*p* = 0.967, Cohen’s d = 0.020, 60 rpm: 59.2 ± 3.9 mL/kg/min, 120 rpm: 59.3 ± 5.5 mL/kg/min). The total workload and achievement rate of the workload calculated before the experiments for each group are shown in Table 2. No significant differences in the total workload and achievement rate of the workload were found between the groups. 

**Figure 1:**
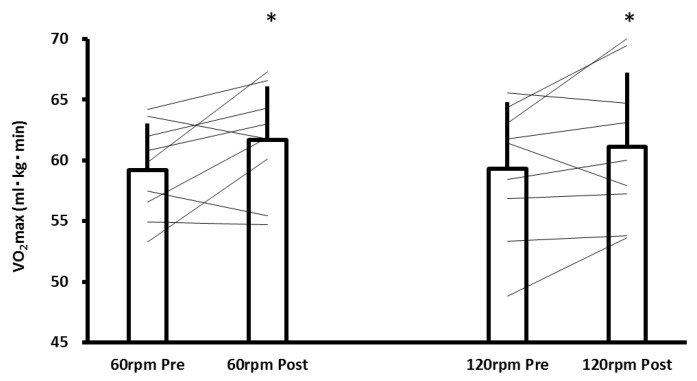
Change of VO_2max_ from pre- to post-training in 60 rpm and 120 rpm. Each line represents the change for an individual participant. Bars are the average value for all participants. Error bars are the standard deviation. * *p* < 0.05 vs pre-training in each group. 60 rpm: high-intensity intermittent cycling training with 60 rpm; 120 rpm: high-intensity intermittent cycling training with 120 rpm.

The main effects of time and interaction were not observed in body weight (main effect of time: *p* = 0.821, partial η^2^ = 0.03, interaction: *p* = 0.821, partial η^2^ = 0.03, 60 rpm pre-training: 65.4 ± 3.9 kg, 60 rpm post-training: 65.4 ± 3.8 kg, 120 rpm pre-training: 64.4 ± 6.3 kg, 120 rpm post-training: 64.3 ± 5.9 kg). Results in terms of change in VO_2max_ from pre- to post-training between groups are shown in Figure 1. The main effect of time was observed in VO_2max_ (*p* = 0.016, partial η^2^ = 0.311). However, no interaction was observed (*p* = 0.680, partial η^2^ = 0.011). No significant difference was detected in the relative change of VO_2max_ (*p* = 0.675, Cohen’s d = 0.201, 60 rpm: 4.3 ± 6.2%, 120 rpm: 3.2 ± 5.5%). The average values of VO_2max_ were as follows: 60 rpm pre-training: 59.2 ± 3.9 mL/kg/min, 60 rpm post-training: 61.7 ± 4.4 mL/kg/min, and 120 rpm pre-training: 59.3 ± 5.5 mL/kg/min, 120 rpm post-training: 61.1 ± 6.1 mL/kg/min.

## 4. Discussion

To the best of the author’s knowledge, this is the first study to examine whether 3-week work-matched HIICT with a high cadence (120 rpm) significantly improves VO_2max_ in university athletes, compared to a low cadence (60 rpm). As a result of a 3-week training period, VO_2max_ increased similarly for 60 rpm and 120 rpm from pre- to post-training. These results were contrary to our hypothesis.

There are two possibilities explaining why there was no significant difference in the effect on VO_2max_ between 60 rpm and 120 rpm in this study. First, the VO_2_ response during the HIICT in this study may be similar for 60 rpm and 120 rpm, unlike the different responses seen in previous studies [15,16,17,18,19,20]. In many previous studies, VO_2_ was higher during work-matched cycling exercise with a high cadence than a low cadence [15,16,17,18,19,20]. However, submaximal exercise intensity was used in these studies, while a much higher supramaximal intensity was used in this study. Recruitment of type II fiber has been shown to be increased at a low cadence compared to a high cadence during submaximal cycling [29,30], and type II fiber has higher ATP consumption than type I fiber [31]. In this study, VO_2_ responses might be similar between 60 rpm and 120 rpm due to the recruitment of more intense type II fibers than in previous studies by using supramaximal intensity. Future studies should investigate in detail the acute VO_2_ response during work-matched HIICT with different cadences.

The second possibility is that workload, rather than exercise intensity, affects VO_2max_. In this study, if VO_2_ during HIICT with a high cadence was higher than with a low cadence like in many previous studies, HIICT with a high cadence was higher in actual intensity compared to that with a low cadence, even though their relative intensity (e.g., %VO_2max_) were the same. Matsuo et al. reported that high-intensity training improves VO_2max_ more significantly than moderate-intensity training due to increased left ventricular mass and stroke volume [21]. On the other hand, Scribbans et al. [32] reported in their meta-analysis that increasing exercise intensity above 60% VO_2max_ does not provide additional increases in VO_2max_. In addition, Granata et al. [23] reported that VO_2max_ can be changed by manipulating the total workload, not the relative intensity. Therefore, the similar chronic effect on VO_2max_ in this study might be related to the equal workload in both groups. 

In this study, the subjects had relatively high initial VO_2max_ levels (60 rpm: 59.2 ± 3.9 mL/kg/min, 120 rpm: 59.3 ± 5.5 mL/kg/min) as compared to previous studies, in which the increase in VO_2max_ was observed following a short training period (32.8–57.3 mL/kg/min) [3,4,5,6,7,8,9]. Nevertheless, VO_2max_ significantly increased in both groups after the 3-week training period. This implies that, for regularly trained athletes, the 3-week HIICT protocol used in this study appears to be an effective method to improve VO_2max_ in the short term, regardless of cadences used during the HIICT.

## 5. Conclusions

We examined whether 3-week work-matched HIICT with a high cadence (120 rpm) significantly improves VO_2max_ in university athletes compared to HIICT with a low cadence (60 rpm). Following a 3-week training period, contrary to our hypothesis, VO_2max_ increased similarly in groups using a cadence of 60 rpm or 120 rpm. These results suggest that cadence during 3-week work-matched HIICT is not training variable affecting the short-term effect of VO_2max_ in university athletes. 

## Figures and Tables

**Table 1:** Average value of the absolute load of high-intensity intermittent cycling training during the training period.

Group	Session	1–2 set (kp)	3–4 set (kp)	5–6 set (kp)	7–8 set (kp)
60 rpm	1–3 session	6.2 ± 0.4	5.9 ± 0.4	5.7 ± 0.4	5.4 ± 0.4
	4–6 session	6.3 ± 0.4	6.1 ± 0.4	5.8 ± 0.4	5.5 ± 0.4
	7–9 session	6.5 ± 0.4	6.2 ± 0.4	6.0 ± 0.4	5.7 ± 0.4
120 rpm	1–3 session	3.1 ± 0.2	2.9 ± 0.2	2.8 ± 0.2	2.7 ± 0.2
	4–6 session	3.1 ± 0.2	3.0 ± 0.2	2.9 ± 0.2	2.8 ± 0.2
	7–9 session	3.2 ± 0.2	3.1 ± 0.2	3.0 ± 0.2	2.8 ± 0.2

VO_2max_ alues are mean ± SD. 60 rpm, High-intensity intermittent cycling training with 60 rpm; 120 rpm, High-intensity intermittent cycling training with 120 rpm.

**Table 2:** Comparisons of total workload and achievement rate during the training period.

	60 rpm	120 rpm	*p* Value	Cohen’s d
Total workload (W)	25,234.7 ± 1572.8	24,897.1 ± 1757.5	0.673	0.202
Achievement rate (%)	98.3 ± 1.0	97.9 ± 1.4	0.522	0.309

Values are mean ± SD. 60 rpm, High-intensity intermittent cycling training with 60 rpm; 120 rpm, High-intensity intermittent cycling training with 120 rpm.
